# Local recurrence of renal cell carcinoma successfully treated with fusion imaging-guided percutaneous thermal ablation

**DOI:** 10.3332/ecancer.2020.1070

**Published:** 2020-07-13

**Authors:** Nicola Camisassi, Giovanni Mauri, Paolo Della Vigna, Guido Bonomo, Gianluca Maria Varano, Daniele Maiettini, Franco Orsi

**Affiliations:** Division of Interventional Radiology, IEO, European Institute of Oncology IRCCS, Milan 20141, Italy; a https://orcid.org/0000-0002-7697-5651

**Keywords:** imaging-guided ablation, renal cell carcinoma recurrence, fusion imaging, radiofrequency ablation

## Abstract

Image-guided thermal ablations are increasingly applied in the treatment of renal cancers, under the guidance of ultrasound (US) or computed tomography (CT). Fusion imaging allows exploitation of the strengths of all imaging modalities simultaneously, eliminating or minimising the weaknesses of every single modality. We present a case of a 68-year-old patient treated using US/CT fusion imaging to guide radiofrequency ablation for local recurrence of renal cell carcinoma undetectable by ultrasound.

## Introduction

Renal cell carcinoma (RCC) is the most common primary malignancy of the kidney, accounting for about 2% of all cancer diagnoses in humans [1]. Image-guided tumour ablation has been used increasingly in the treatment of patients with renal tumours, particularly in the case of lesions smaller than 4 cm, and in patients with single kidney with the aim of avoiding loss of renal function deriving from surgical nephrectomy [2]. The availability of the most advanced imaging techniques, including a dedicated room with computed tomography (CT) and a last generation ultrasound (US) machine, possibly equipped with fusion imaging, may allow the correct targeting of tumour not well visible by ultrasound alone and thus to maximise the technical result [3, 4]. We here present a case of a 68-year-old patient with previous nephrectomy successfully treated using US/CT fusion imaging to guide radiofrequency (RF) ablation for local recurrence of RCC undetectable by ultrasound.

## Case report

**Figure 1.**
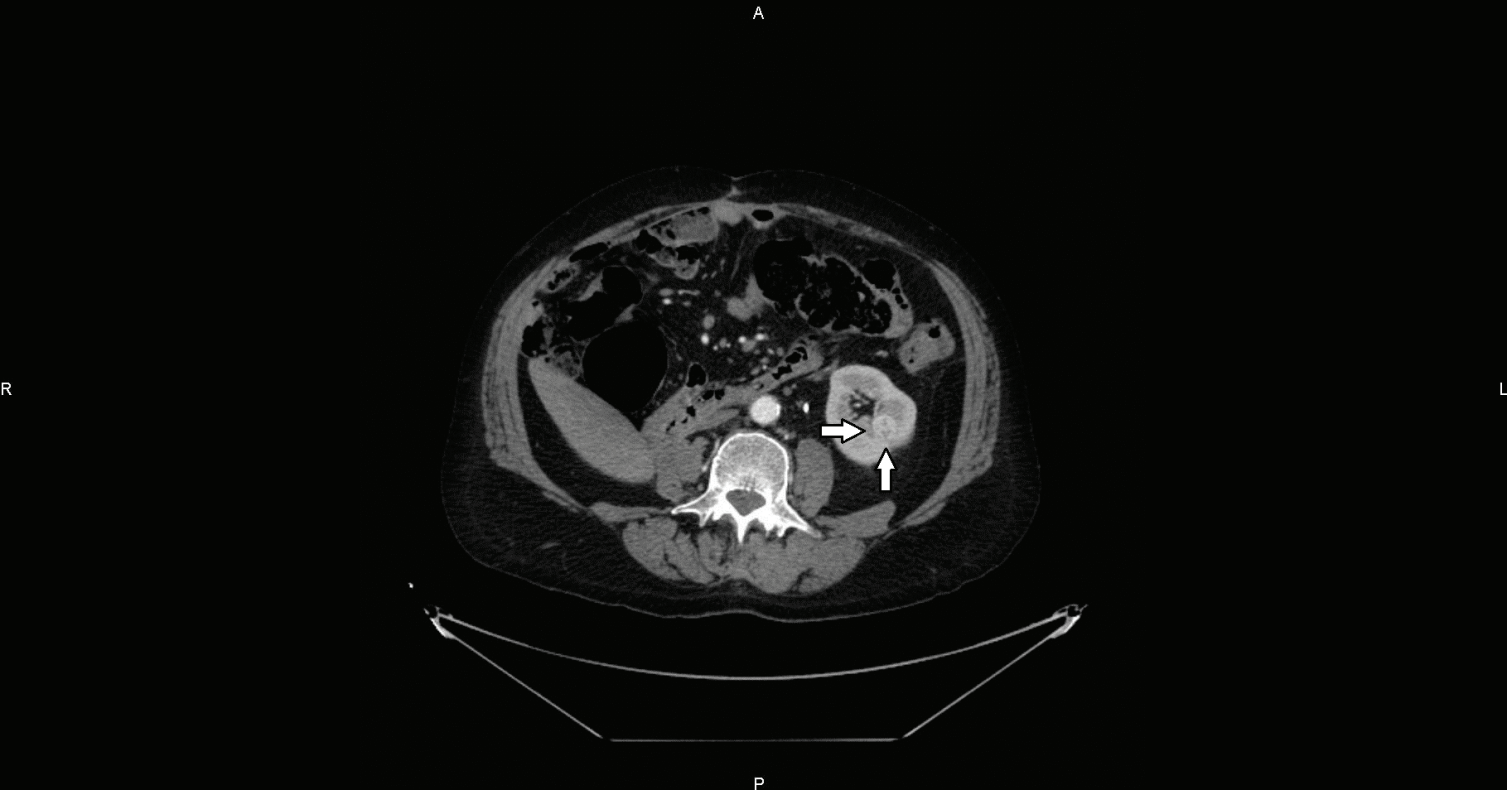
Axial CT image shows an intraparenchymal mid-renal hypervascular nodule referred to RCC (arrows).

**Figure 2.**
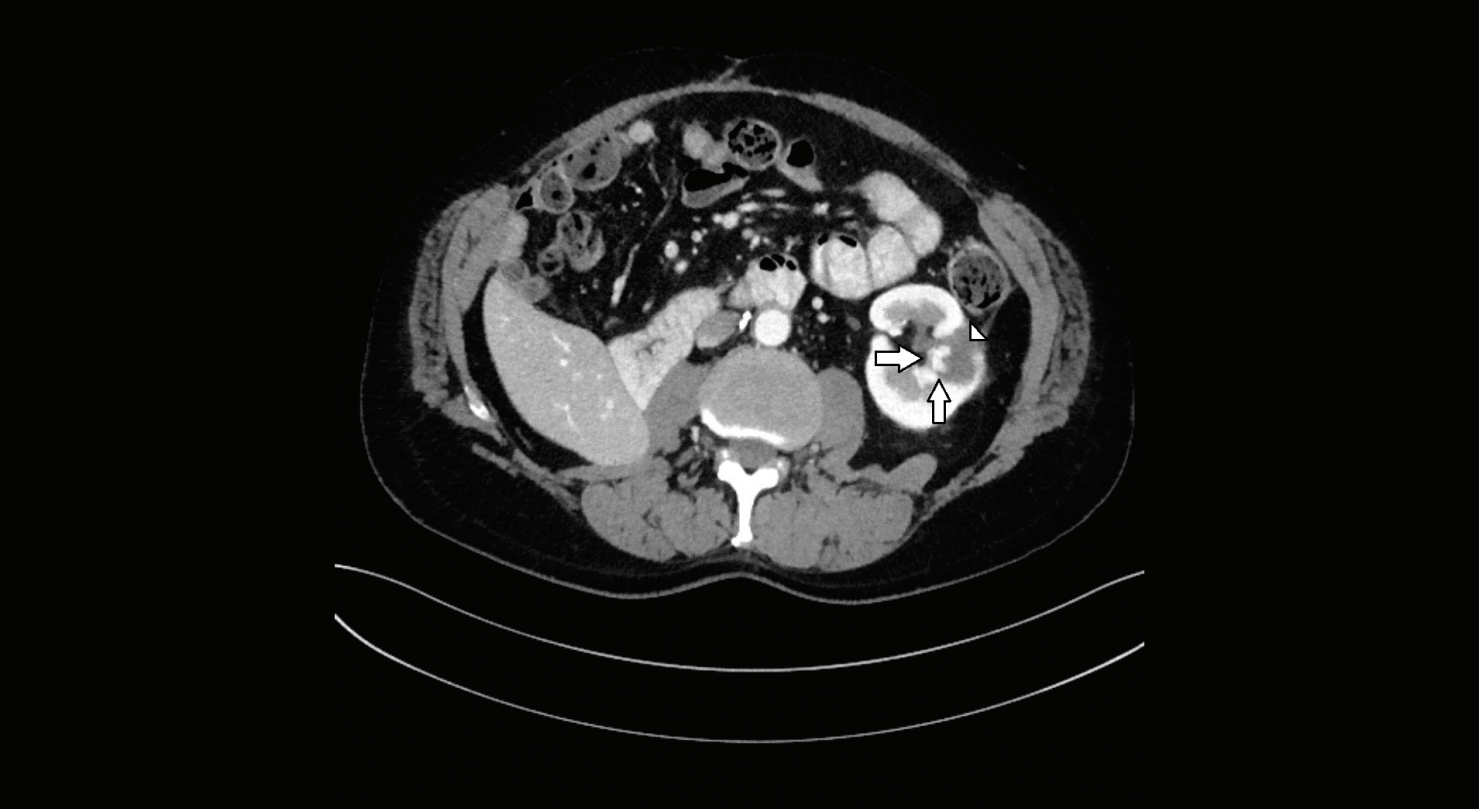
Axial CT image shows the hypervascular nodule (arrows), centrally located, on the inner margin of the previous ablation area (head of arrow).

**Figure 3.**
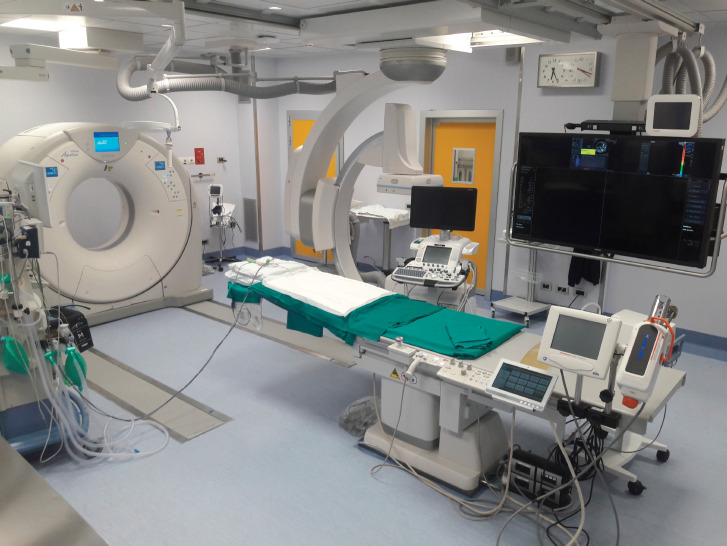
Angio-CT Hybrid Suite with the availability of angiography, CT and US.

**Figure 4.**
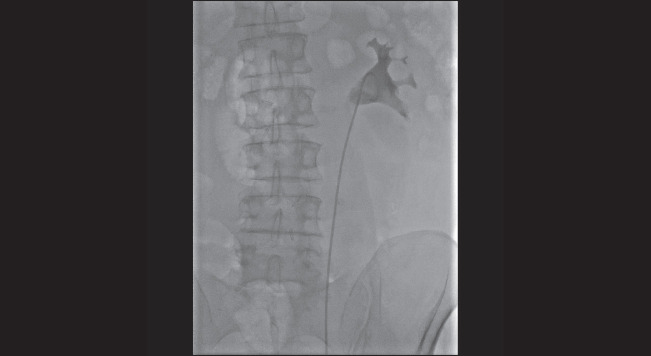
Retrograde pyelography shows the right positioning of the left ureteral stent in the omolateral collecting system.

**Figure 5.**
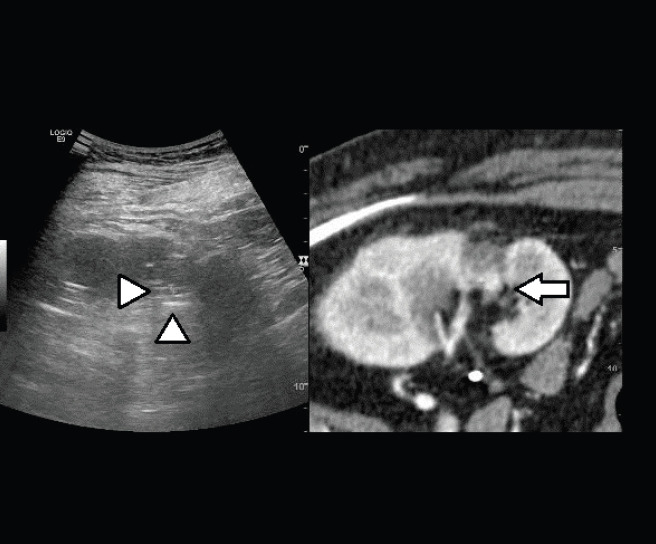
Fusion imaging combining real time US with CT images: CT scan shows the hypervascular nodule deeply in the scar of the previous treatment (arrow); the lesion is not clearly visible at US (heads of arrow).

**Figure 6.**
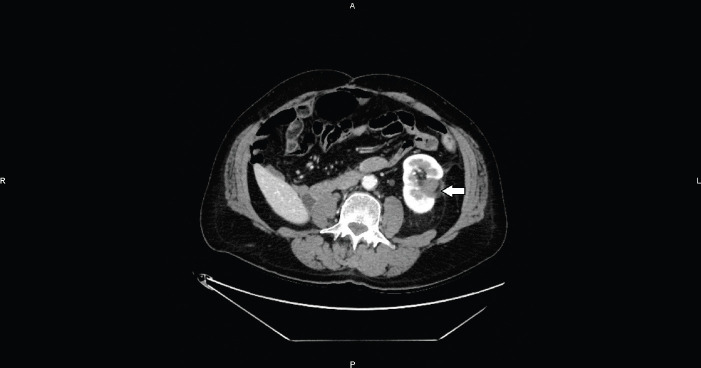
Axial CT image shows the hypo-enhancing ablation zone without enhancing residual tumor (arrow).

A 68-year-old-male patient with a history of right nephrectomy was treated 3 months before by microwave ablation for RCC of the left kidney (Figure 1). He presented with local recurrence and was referred to our institution for treatment. Multi-detector computed tomography (MD-CT) showed the presence of a 15 mm hypervascular nodule, centrally located, on the inner margin of the previous ablation area (Figure 2). Despite the recurrence after the first treatment, surgery was still considered too risky since his solitary kidney so, after multidisciplinary discussion and agreement, another attempt of imaging-guided ablation was proposed as first line treatment. It was decided to use RF ablation combined with pyeloperfusion, in order to protect the collecting system from thermal damage. The procedure was performed under general anaesthesia in our Angio-CT Hybrid Suite with the availability of angiography, CT and US (Figure 3). First a 6 French stent (Pollack Open-End Flexi-Tip® Ureteral Catheter, Cook Urological) was placed into the ipsilateral renal pelvis (Figure 4). Then, we preliminarily performed an US examination to localise the lesion, but unfortunately, it was completely invisible on B-mode US due to its central location in the kidney, completely hidden behind the scar of the previous treatment. With patient in right lateral decubitus, MD-CT with contrast media was performed and data were transferred in DICOM format to the US system. Fusion imaging was used to guide the needle to target the residual tumour combining real-time US scans with CT images (Figure 5). Radiofrequency ablation (RFA) was performed with 3 cm umbrella-shaped multi-tines needle electrode (LeVeen CoAccess RFA needle electrode, Boston Scientific, MA, USA), selected to match the size of the tumour. The electrode was inserted and then connected to the generator (RF 3000, Boston Scientific) after checking the correct positioning with a CT scan; energy was applied till roll-off occurred. Roll-off was achieved three times (80 W at 11 min, 60 W at 8 minutes and 60 W at 10 minutes) before needle removal. During ablation, cold pyeloperfusion was performed with gentle injection of sterile water through the ureteral stent. Patient was discharged the day after in good conditions after a clinical evaluation and MD-CT performed to exclude complications. Follow-up CT scan performed at 6 weeks showed complete tumour ablation without enhancing residual tumour (Figure 6). Renal function did not significantly change at 6 months from the treatment (preoperative creatinine levels = 1.65 mg/dl, 6 months creatinine levels = 1.63 mg/dl)

## Discussion

RCC accounts for 2%–3% of all adult malignancies [5]. The detection of RCC is increasing owing to an increase in the diagnosis of small asymptomatic renal masses with cross-sectional imaging [5]. Radical nephrectomy represents the conventional treatment, while, more recently, surgical nephron sparing techniques have been developed with the aim of reducing invasiveness [6–8]. The application of image-guided ablative techniques to renal cancer has pushed in order to offer treatment to patient not suitable for surgery and to spare the highest amount of renal parenchyma [6, 9, 10] with reported good clinical results [11, 12]. The most widely used ablative techniques used to treat renal tumours are cryoablation, RF ablation and microwave ablation [13–15]. With RF an alternating current determines ionic friction that leads to slow heat generation, with subsequent protein denaturation, blood coagulation and coagulative necrosis [16, 17]. US is the most commonly used image guidance method worldwide to guide percutaneous thermal ablation due to its low cost, availability, high contrast resolution, absence of ionising radiation and real-time guidance capabilities in any imaging plane [18, 19]. However, it can be sometime difficult to distinguish between simple and complex or neoplastic cyst and to detect and characterise solid lesions so that a second level method such as CT or MRI is often required [20]. In case of lesions undetectable with ultrasound, imaging-guided ablation is often considered to be not feasible. In those cases, real-time fusion imaging of ultrasound and pre-acquired CT or MRI images has been reported as a feasible and an effective way to successfully detect the target lesion and perform percutaneous ablations [18, 21]. In a study of Auer *et al* [22] fusion imaging, combining real-time US and pre-acquired CT or MRI images, were performed successfully in the majority (89.3%) of patients in which either a kidney lesion of interest could not definitively be localised or sufficiently defined with grey-scale US alone. Contrast-enhanced ultrasound is also emerging as a useful technique to study cystic lesions and their related septal vascularisation, as well as solid lesions, during ablative procedures [23, 24]. However, Helck *et al* [25] have already described a more accurate identification of kidney lesions with fusion imaging compared to US and CEUS alone. When targeting renal lesions during imaging-guided ablation, fusion imaging can help in recognising the most appropriate part of the lesion to biopsy (especially, for cystic lesions) and distinguishing more precisely its margins in order to find the best position to place the electrodes for ablation, as in our case. Furthermore, the use of fusion imaging is valuable in determining the correct path for the needle, avoiding harm to structures such as the renal vessels, renal pelvis, adrenal glands, spleen and colon. In the case presented, it would have been not possible to treat the patient with thermal ablation without the application of fusion imaging, and the patient would have been treated by surgical nephrectomy, with consequent loss of renal function and need of dialysis for the rest of his life. With the application of fusion imaging in our dedicated hybrid angio suite, it has been possible to successfully treat the patient with RF ablation, with limited impact on renal function.

## Conclusion

Especially when treating residual or recurrence of renal lesions, either after surgery or percutaneous thermal ablation, tumour could not be sufficiently defined with grey-scale US alone. The combination of real-time US scans with CT images is an essential tool to place applicators for ablation, thus offering to the patients a chance to be treated in a minimally invasive way.

## Conflicts of interest statement

The authors declare that they have no conflicts of interest.

## Funding declaration

The authors received no specific funding for this work.
